# Post-translational modification of NK cell receptors offers clues to antigenic specificity riddle

**DOI:** 10.3389/fimmu.2025.1671844

**Published:** 2026-01-07

**Authors:** Maria O. Ustiuzhanina, Elena I. Kovalenko, Dmitry M. Chudakov

**Affiliations:** 1 Center for Molecular and Cellular Biology, Moscow, Russia; 2 Shemyakin and Ovchinnikov Institute of Bioorganic Chemistry, Russian Academy of Sciences, Moscow, Russia; 3 Institute of Translational Medicine, Pirogov Russian National Research Medical University, Moscow, Russia; 4 Abu Dhabi Stem Cell Center, Abu Dhabi, United Arab Emirates

**Keywords:** adaptive NK cell clones, antigen-specific NK cells, KIR, NK cell memory, viral infections

## Abstract

Natural killer (NK) cells protect against infections through a distinctive combination of innate and adaptive immune capabilities. They exhibit characteristics of immunological memory by enhanced secondary response to pathogen exposure. We discuss current progress in identifying long-lived NK cell clones with enhanced memory functionality and the capacity for intensive cytokine and cytotoxic granule production upon re-encountering external antigens. We examine data related to how various NK cell receptors facilitate the recognition of specific foreign peptides in particular human leukocyte antigen (HLA) contexts and may promote the formation of memory clones. Finally, we propose and substantiate a model that resolves the accumulated fundamental contradictions and explains the semi-antigen-specific nature of the NK cell response through the clonally imprinted expression patterns of enzymes involved in the post-translational modification of HLA-binding receptors.

## Introduction

Accumulated data suggest that specific subsets of NK cells possess characteristics of adaptive immunity, sharing many features with the clonal organization and memory responses typical of T cell immunity (1–3). NK cells develop from the same common lymphoid progenitor (CLP) that gives rise to T and B cells (4, 5), and like these cells, NK cells require common gamma-chain cytokines, such as IL-15, for their development and homeostasis (6, 7). Similar to T cells, a combination of signals is required for the activation of memory processes in NK cells, including auxiliary activating signals from other receptors and stimulation by cytokines such as IL-12 or type I interferons (8–10).

NK cell sensitivity is tuned through “education”, a process in which their responsiveness is programmed via interaction between receptors, including members of the killer immunoglobulin-like receptor (KIR) family and natural killer group 2 (NKG2), and self-ligands, typically MHC-I (11–13). This is somewhat analogous to T cell maturation in the thymus (10), where the latter undergo screening and selection to achieve proper functionality and self-tolerance of the naive T cell repertoire.

The defining characteristic of adaptive immunity is immunological memory, which refers to the ability of previously activated immune clones to persist in the body for extended periods and rapidly and efficiently expand upon re-encountering the activating antigen. Innate immune cells can also exhibit altered responses in the aftermath of initial stimulus that shapes their behavior upon repeated exposure to the same stimulus, called trained immunity (14–17). Phenomenon of trained immunity may be also associated with clonal proliferation of innate immune cells (15, 18), classically described in monocytes/macrophages (19, 20). It has been proposed that alterations in DNA methylation-dependent pathways may underlie the long-lasting phenomenon of trained immunity, which largely brings trained immunity and immunological memory closer in terms of mechanisms (21).

Memory-like responses of NK cells were established to be induced by cytokine stimulation (22–25), CD16a activation (26), LPS stimulation (27), and tumor-priming (28). Furthermore, NK cell memory may encompass recallable, cell-intrinsic enhancements that demonstrate features of antigenic specificity. Effector CD8^+^ T cell clones rely on their T cell receptors to recognize specific foreign epitopes presented by MHC-I molecules (29). Distinct and specific response of NK cells is essentially dependent on the combinatorial code of activating and inhibitory receptors including those that bind to HLA class I molecules. At the same time, the interaction between NK cell receptors and target cells depends not solely on the presence of a particular MHC-I variant on a target cell but also on the specific peptides presented within the MHC-I binding groove (30), which can affect the affinity and stability of the interaction, influencing NK cell response (30, 31). Several studies have explicitly shown that certain NK cell receptors preferentially bind classical and non-classical MHC-I molecules presenting specific viral peptides (pMHC) (32–35). Apart from MHC-I specificity, NK cell receptors may directly recognize some viral antigens (36).

Some of the earliest evidence for the potential of NK cells to function as memory cells came from experiments with *Rag2*-deficient mice and was based on their response to 2,4-dinitrofluorobenzene and oxazolone after undergoing previous sensitization with haptens (37). Further, adaptive functions of NK cells were established in the context of mouse cytomegalovirus (mCMV) infection (1). Ly49H^+^ NK cells exhibited phases of immune response against the spread of the pathogen that resembled adaptive immune memory (1, 10, 38). NK cells are known to play an important role in immune defense against human CMV (hCMV) as well (39–41), and a study in humans identified a distinct population of NKG2C^+^ NK cells associated with hCMV infection that exhibit properties of immunological memory (42). A series of studies including our work addressed the ability of NKG2C^+^ NK cells to specifically recognize the peptide, derived from hCMV protein UL40 and UL120 (43–46). Significant similarity of the processes of memory formation in T and NK cells was confirmed by a similar pattern of epigenetic reprogramming (47–49). Recent high-dimensional single-cell study identified an ‘NK3’ cluster in human peripheral blood that closely mirrors adaptive NK cells, capturing their transcriptional program and epigenetic imprinting and appearing across donors irrespective of hCMV status (47).

Recent studies have revealed that under CMV infection, memory NK cell populations in both mice and humans can arise following clonal expansion (50). In mice, the clonal nature of memory NK cells was highlighted by an innovative approach using retroviral barcoding combined with fluorescent protein labeling to track individual Ly49H+ NK cells. Upon transfer into immunodeficient recipients followed by infection with mCMV, these barcoded Ly49H+ NK cells underwent dramatic expansion, with some clones generating progeny exceeding 10,000 cells at the peak of the response (51). In humans, an imprinted pattern of particular self-specific activating KIRs (KIR2DS4, KIR2DS2 or KIR3DS1) indicated clonal-like expansion of human adaptive NKG2C+ NK cells (52). More insights have emerged through the application of mitochondrial DNA mutation tracking, a technique previously used to trace clonal lineages (53), Rückert et al. demonstrated that many of the large scATAC-Seq clusters actually represent clonal NK cells (54). These studies highlight the capacity of NK cells to form clonal populations, a feature that aligns them more closely with adaptive immune cells and emphasizes their role in long-term immune defense. However, the mechanisms of selection leading to the production of clonal and furthermore, relatively antigen-specific NK cells, despite significant progress in this area, are still far from being understood.

Here, we overview the current knowledge on the clonal nature of NK cell memory and peptide-dependent recognition by NKG2 and KIR receptors in immune responses to viral and bacterial infections. To resolve the fundamental contradiction between the innate nature of NK cell receptors and the adaptive immunity features of NK clonal memory, we propose a hypothesis that may reconcile the existing inconsistencies, supported by *in silico* modeling and single-cell RNA sequencing data analysis.

## Peptide-dependent NK cell interaction with HLA-I

**Figure 1:**
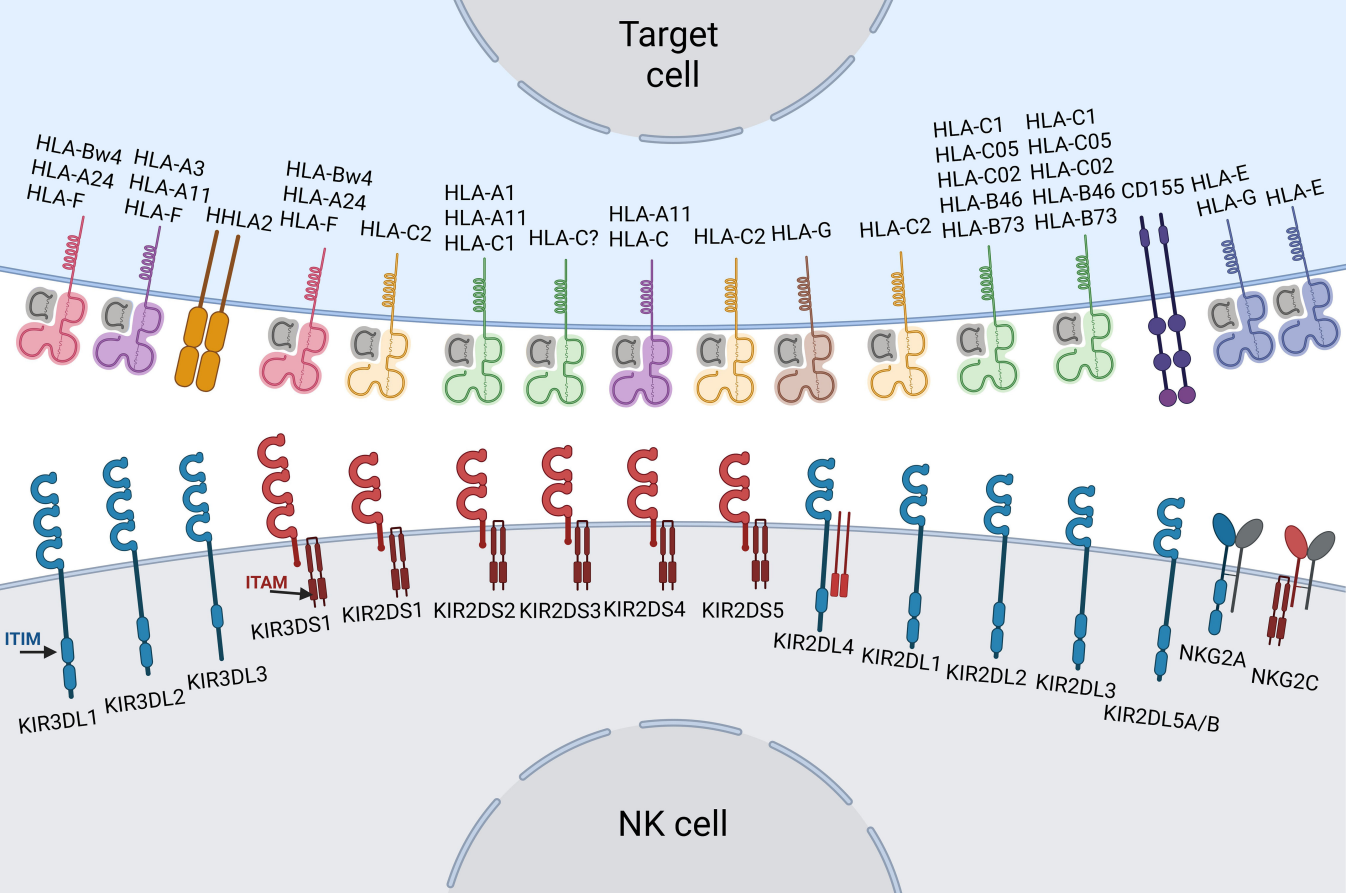
Schematic of major interactions between KIRs, NKG2A, NKG2C and HLA molecules. The eight inhibitory KIRs (KIR2DL5A and KIR2DL5B are shown as a single grouped entity (KIR2DL5A/B)) and NKG2A are colored blue, the six activating KIRs and NKG2C are shown in red. Each IG-like domain is depicted as a semicircle. The cytoplasmic tails of the inhibitory KIRs include ITIM motifs (blue ellipses). The short-tailed activating KIRs carry a positively-charged amino acid residue in the transmembrane region that enables interaction with the DAP-12 adaptor protein, which contains ITAM motifs (dark red rectangles). HLA-I variants are colored as follows: HLA-C2 = yellow, HLA-C1 = green, HLA-Bw4 = pink, HLA-A = purple, HLA-E = indigo. Specific alleles are written above the ligand. Note that each NK clone expresses and carries only a subset of KIR molecules, determined by stochastic epigenetic imprinting prior to NK cell education.

The expression pattern of HLA-I molecules on target cells is of decisive importance for the functional activity of NK cells, and the outcome of those interactions is shaped by their multiple inhibitory and activating receptors (**Figure 1**) (55). Here, we focus on two main receptor families that can interact with HLA-I (MHC-I in humans): natural killer group 2 (NKG2) receptors and KIRs (**Table 1**).

**Table 1:** HLA-related NK cell receptors.

Action	Family	Receptor	Ligand	Reference
Inhibitory	NKG2	NKG2A	HLA-E, HLA-G	(56, 57)
		NKG2B	HLA-E	(56)
	KIR	KIR2DL1	HLA-C2	(58)
		KIR2DL2	HLA-C1	(58, 59)
		KIR2DL3	HLA-C1	(58)
		KIR2DL5A/B	CD155	(60)
		KIR3DL1	HLA-A*24, HLA-Bw4, HLA-F	(61–63)
		KIR3DL2	HLA-A*3, HLA-A*11, HLA-F	(62, 64)
		KIR3DL3	HHLA2	(65)
Activating	NKG2	NKG2C	HLA-E	(56)
		NKG2D	MICA, MICB…	(66)
		NKG2E	–	–
		NKG2F	unknown	–
		NKG2H	unknown	–
	KIR	KIR2DS1	HLA-C2	(58)
		KIR2DS2	HLA-C1, HLA-A	(67)
		KIR2DS3	unknown	–
		KIR2DS4	HLA-C, HLA-A*11	(68, 69)
		KIR2DS5	HLA-C2	(70)
		KIR3DS1	HLA-F	(62)

### NKG2 family

NKG2 receptors are type II transmembrane proteins containing a lectin-like C-type extracellular domain (71). There are seven NKG2 molecules; five of these (NKG2A, NKG2B, NKG2C, NKG2E, and NKG2H) are associated with CD94, NKG2D form homodimers, whereas the information about NKG2F is limited (72). The genes encoding the NKG2 receptor family reside on human chromosome 12p13, in a ~2.5 Mb region called the natural killer complex (NKC) (73). NKG2A and NKG2B are inhibitory NK cell receptors; they contain two immunoreceptor tyrosine-based inhibitory motifs (ITIM) responsible for signal transduction (74). The primary ligand for those receptors is HLA-E, a non-classical class I molecule (56). NKG2A has also recently been shown to bind to HLA-G (57). NKG2C, NKG2E, and NKG2H are considered to be activating receptors of NK cells. Together with CD94 and the DAP12 adapter protein, they can form large complexes that, when bound to a ligand, transmit activating signals to NK cells (75). HLA-E is also the primary ligand for activating CD94/NKG2 receptors (56). NKG2E and NKG2H are the least studied NKG2 receptors. NKG2E has never been found on the cell surface and is instead considered to be an intracytoplasmic protein (76). In contrast, NKG2H is expressed on the cell surface, but is more commonly found on T cells than NK cells (77). Unlike most other NKG2 receptors, activating NKG2D receptor does not dimerize with CD94, and associates with the transmembrane domain of the DAP10 adapter protein (78). The primary ligands of NKG2D are the stress-inducible non-classical MHC molecules polypeptide-related sequence A (MICA) and B (MICB) with considerable allelic diversity (79). Structurally, MICA and MICB are similar to MHC I molecules, but they do not include ß2-microglobulin and are unable to present peptides (66).

The interaction between NKG2 family members and HLA-E ligands occurs after HLA-E has been loaded with 9-mer peptides, which are processed out of endoplasmic reticulum (ER) leader peptides derived from HLA-A, -B, -C, and -G (56, 71). Once stabilized by the leader peptide, HLA-E reaches the cell surface and binds to NKG2A, generating inhibitory signals in NKG2A-positive NK cell clones (46, 71, 80). As a heterodimer, the NKG2A/CD94 complex forms a binding interface with HLA-E through the connection of CD94 with the α1 helix and NKG2A with the α2 helix of HLA-E (81). Notably, the binding of NKG2A to HLA-E involves direct interaction between NKG2A and the presented peptide (82). Consequently, the affinity and strength of the inhibitory signal generated by NKG2A depends on which peptide loaded onto HLA-E, the dissociation constant ranged from 0,18 to 3 µM (71, 82–84). Significant inhibitory signals were associated with the HLA-G leader peptide (80, 83). The affinity of NKG2C for peptide-loaded HLA-E binding is ~5-fold lower than that of NKG2A (71, 84, 85). Furthermore, the peptide specificity of NKG2C interaction with HLA-E is even stronger than for NKG2A; only four out of 14 tested peptides were able to activate NKG2A^-^NKG2C^+^ NK cells (86, 87). Recently, a wide range of peptides derived from endogenous proteins was found to stabilize HLA-E surface expression and modulate NK cell functionality (46). Consequently, the difference in peptides presented by HLA-E molecules may affect the NK cell response.

### KIR family

KIRs are the most polymorphic class of NK cell-surface receptors. They belong to the immunoglobulin superfamily and are structurally characterized by two or three extracellular immunoglobulin (IG)-like domains and a short (S) or long (L) intracytoplasmic region that can transmit either activating or inhibitory signals. KIRs are encoded in the leukocyte receptor complex (LRC) on chromosome 19q13.4 and are characterized by a high degree of variability due to differences in KIR genes, their copy number, and allelic polymorphisms observed at each locus (88). There are 14 functional KIR genes and two pseudogenes, KIR2DP1 and KIR3DP1. The six KIR genes with S intracytoplasmic regions encode activating KIRs, while the nine genes with L regions encode inhibitory KIR receptors and KIR2DL4, which can perform both activating and inhibitory functions (89). There are two broad KIR gene haplotypes, A and B, which are divided into centromeric and telomeric regions. The framework genes, presented in all individuals, are KIR2DL4, KIR3DL2, and KIR3DL3 (90, 91). Haplotype A includes 3 inhibitory KIRs (KIR2DL1, KIR2DL3, KIR3DL1) and KIR2DS4; haplotype B includes as well KIR2DL1; allelic variants of KIR2DL3 (KIR2DL2), KIR3DL1 (KIR3DS1), KIR2DS4 (KIR2DS1); and other activating and inhibitory receptors (KIR2DL5, KIR2DS2, KIR2DS3/5) (88, 92). Both haplotypes feature variable genes; the most variable genes are in the telomeric half for A haplotype and in the centromeric half for B haplotypes (93, 94). Haplotype B is more variable than haplotype A and accounts for more than 40 distinct haplotypes (95). Currently, the total population diversity of KIR alleles is estimated to include ~2,219 alleles (96).

KIRs interact with both classical and non-classical HLA-I-peptide complexes (**Tables 1**, **2**). HLA-I molecules exhibit significant polymorphism, and distinct KIRs exhibit selective interactions with specific HLA-I alleles. In the course of NK cell education process, which may resemble T cell thymic selection (110), clonally-expressed KIRs bind to self HLA-I molecules (111), and the balance of inhibitory and activating signals dictates survival and maturation of each NK clone. Some KIR receptors can recognize HLA-C molecules, where recognition is dependent on the amino acid at position 80 in HLA-C: asparagine for the C1 epitope, and lysine for the C2 epitope (112). KIR2DL2/DL3 and KIR2DS2 bind HLA-C1, while KIR2DL1, KIR2DS4, and KIR2DS1 bind HLA-C2 (59, 67, 68, 113). KIR2DL2/DL3 can also bind to some HLA-C2 alleles (HLA-C*05:01 and HLA-C*02:02) as well as HLA-B*46:01 and B*73:01 (114, 115). It has also been shown that KIR2DL2 binds to HLA-C2 with higher affinity than KIR2DL3 (114). The affinity of a given KIR for HLA-C is informed by the alleles for both genes. KIR2DL1 allelic variant of haplotype A centromeric region (CenA) strongly binds to HLA-C2 receptors, while allelic variant of haplotype B centromeric region (CenB) weakly binds to HLA-C2. Similarly, KIR2DL3 variants from CenA region weakly bind to HLA-C1, while KIR2DL2 allelic variants from CenB region strongly bind to HLA-C1 (58). Other KIRs are able to interact with HLA-B and -A molecules. KIR3DL1 and KIR3DS1 bind HLA-Bw4, a variant with an isoleucine residue at position 80 (116). Interestingly, KIR3DL1 displays the highest number of allelic variations, which impacts its affinity for HLA-Bw4 (117). For instance, KIR3DL1*002 is a stronger inhibitory receptor for HLA-Bw4 compared to KIR3DL1*007 (61). Additionally, some KIRs bind to non-classical HLA-I; KIR3DS1, KIR3DL2, and KIR3DL1 interact with HLA-F in descending order of affinity (62).

**Table 2:** Interaction of NK cell receptor with peptide in the context of HLA-I.

NK cell receptor	HLA molecule	Peptide	Source of peptide	Outcome	Refs
KIR2DS1	HLA-C*06:02	SRGPVHHLL	Synthetic peptide	activation	(97)
	HLA-Cw4	QYDDAVYKL	Consensus peptide	activation	(30, 98)
	HLA-C*05:01	IIDKSGAWV	Human modified	NT	(99)
		IIDKSGAVV	Human modified	NT	
		IIDKSGIPV	Human modified	NT	
KIR2DS2	HLA-C*01:02	LNPSVAATL	HCV, helicase	activation	(34)
		IVDLMCHATF	ZIKV and DENV, genome polyprotein	activation	
	HLA-A*11:01	MLIYSMWGK	VACV, MpA14	activation	(100)
KIR2DS4	HLA-C*05:01	IIDKSGAWV	Human modified	activation	(68)
		IIDKSGAFV	Human modified	activation	
		IIDKSGAYV	Human modified	activation	
		SNDDKNAWF	Human	activation	
		IVDKSGAWF	*Campylobacter jejuni*, RecA	activation	
		IIDKKGSWF	*Chlamydia trachomatis*, RecA	activation	
		VVEKSGAWF	*Brucella abortus*, RecA	activation	
		IVDKSGAWL	*Helicobacter pillory*, RecA	activation	
		VIEKAGSWF	*Thermus aquaticus*, RecA	activation	
KIR3DS1	HLA-B*57:01	AAVKAACWW	HIV, Gag-Pol	activation	(101)
		KAAFDLSFF	HIV, Nef	activation	
		LSSPVTKFF	Human modified	NT	
KIR2DL1	HLA-Cw4	QYDDAVYKL	Consensus peptide	NT	(98, 102)
	HLA-C*05:01	IIDKSGAWV	Human modified	NT	(99)
		IIDKSGAVV	Human modified	NT	
		IIDKSGIPV	Human modified	NT	
KIR2DL2	HLA-C*07:02	RYRPGTVAL	Human, histone H3	NT	(103)
	HLA-C*03:04	GAVDPLLAL	Human, importin subunit	NT	(59, 103)
KIR2DL3	HLA-C*03:04	YIPLVGAPL	HCV, core protein	inhibition	(104)
		VIYPARISL	Human	inhibition	(35)
		YAIQATETL	HIV-induced Human	no inhibition	
		GAVDPLLAL	Human, importin subunit	NT	(59, 103)
	HLA-C*07:02	RYRPGTVAL	Human, histone H3	NT	(103)
KIR3DL1	HLA-B*27:05	KRWIILGLNK	HIV, p24 Gag	NT	(105)
	HLA-B*57:01	ISPRTLNAW		NT	
	HLA-B*57:03			NT	
	HLA-A*24:02	TYQWIIRNW	Influenza, Pol basic protein	NT	(106)
		FYRYGFVANF	Influenza, RNA-directed RNA pol catalytic subunit	NT	
		RYGFVANF		NT	
		RYPLTFGW	HIV, Nef	NT	
KIR3DL2	HLA-A*03:01	RLRAEAQVK	EBV, EBNA3A	NT	(64)
	HLA-A*11:01				
NKG2C	HLA-E	AISPRTLNA	HIV, Gag	activation	(107)
		VLKYWWNLL	HIV, Env	activation	
		ILPCRIKQI	HIV, Env	activation	
		TMDSNTLEL	Influenza	activation	
		VMAPRTLFL	hCMV, UL40 Human, HLA-G	activation	(43)
		VLPHRTQFL	hCMV, UL120	activation	(46)
		multiple	Human	NA	
NKG2A	HLA-E	VLPHRTQFL	hCMV, UL120	inhibition	(46)
		multiple	Human	NA	
		GGDPHLPTL	EBV, LMP1	activation	(33)
		VMPLSAPTL	SARS-CoV-2, Nsp13	activation	(108)
		VFLVLLPLV	SARS-CoV-2, spike	inhibition	(109)

KIRs directly interact with peptides presented by HLA-I, influencing binding affinity (59, 98, 100, 103). For example, KIR2DS1 and KIR2DL1 bind HLA-Cw4 (HLA-C2 group) presenting the QYDDAVYKL peptide (30, 98). However, substitutions in amino acids at positions 7 and 8 of the peptide abolish this interaction (30, 118). Similarly, the interactions between KIR2DS2/KIR2DL3/KIR2DL2 and HLA-Cw3 (HLA-C1 group) are peptide-dependent, and the strength of binding increases in this line (30, 119). In a subsequent test of 28 self-peptides presented in HLA-C*08:02, only 13 and 11 peptides, respectively, were able to interact with KIR2DL2 or KIR2DL3. An analysis of these data showed that the presence of serine or threonine at position 8 of the peptide was preferable for KIR interaction, whereas positively-charged residues appear to be unfavorable at this position (31). Binding of soluble version of KIR2DL1 to HLA-C*05:01 was less selective, with 24 out of 28 peptides tested forming a stable interaction (31). Even more, the allelic polymorphism of KIRs affects their interaction with a peptide; the self-peptide ASLNLPAVSW presented in HLA-B*57:03 form contacts with KIR3DL1*114 and *086, while no contacts were found with KIR3DL1*001, resulting in a 25 times higher dissociation constant (106, 120).

The binding of activating KIRs is seemingly more peptide-specific. For KIR2DS1 interaction with HLA-C*06:02 loaded with 20 different peptides, binding only occurred in the presence of one synthetic peptide (SRGPVHHLL), activating KIR2DS1^+^ cells (97). KIR3DS1 binding is dependent on the amino acid at position 8 of peptides presented by HLA-B*57:01; KIR3DS1 did not bind to LSSPVTKSF in this context but bound strongly when the Ser at position 8 was substituted with Phe (LSSPVTKFF) (101). KIR2DS4 binding to HLA-I is highly specific: none of 46 self-peptides from HLA-C*05:01 conferred binding. Substitutions in different amino acids of one of these self-peptides (IIDKSGSTV) showed that KIR2DS4 can bind to HLA-C*05:01 presenting peptides that contain Ala at position 7 and Trp at position 8 (IIDKSGAWV). The authors proposed that aromatic rings at position 8 may contribute to the binding process; the presence of Phe or Tyr at position 8 also facilitated KIR2DS4 binding to HLA-C*05:01, while Val prevented the interaction. However, in an analysis of 12 other self-peptides with Trp at position 8 presented by HLA-C*05:01, only SNDDKNAWF allowed binding to KIR2DS4 (68).

The presented receptors of KIR and NKG2 groups are possibly involved in the recognition of viral and bacterial peptides, during interaction with HLA-I molecules and may play a pivotal role for the regulation of NK cell activity and memory formation.

## Memory NK cell clones defend against infections

### Influenza

NK cells produce IFNγ in response to influenza virus, which magnitude depends on the strain, with the highest levels observed for the California H1N1 strain (107, 121). IFNγ production by NK cells persists four weeks after vaccination, and this may represent a memory-like response (122). Accordingly, high IFNγ release was observed in vaccinated patients stimulated with inactivated influenza H1N1 strain compared to unvaccinated patients (122). The family of activating natural cytotoxicity receptors (NCRs), which includes NKp30, NKp44, and NKp46, is an important component of the NK response to influenza (123). NKp46^+^ NK cells can bind to viral hemagglutinin and eliminate cells infected with H1N1 influenza (36, 124), and mouse studies have shown that NKp46^+^ NKG2A^+^ NK cells recognize glycosylation sites in hemagglutinin protein, leading to increased IFNγ production (125). However, the formation of memory-like NK cells could occur without the presence of hemagglutinin protein. For example, virus particles containing influenza matrix protein 1 can induce NK cell sensitization and memory formation (126). This may suggest that additional NK cell receptors are involved in recognizing the influenza virus.

Previous studies have shown that the HLA-binding NKG2C and KIRs can impact NK-mediated immune defense against influenza. Vaccination leads to an increase in NKG2C^+^ NK cells in responsive donors, with high titers of antibodies against hemagglutinin protein (127). Clonal NK cells derived from healthy donors specifically lysed HLA-E-expressing K562 cells loaded with the TMDSNTLEL peptide from the H1N1 influenza virus. Blocking of NKG2C significantly reduced the degranulation of these clonal NK cells when exposed to this peptide (107). Associations of immune response with KIR/HLA-I pairs are shown. KIR2DL3^+^ NK cells, which are known to interact with HLA-C1, responded to purified H1N1 more rapidly in terms of IFNγ production and showed elevated CD107a degranulation profile compared to KIR2DL1^+^ NK cells interacting with HLA-C2 and KIR3DL1 with HLA-Bw4 (128). However, an analysis of intensive care unit patients showed fewer deaths from influenza (H1N1) among KIR3DL1/S1^+^ patients compared to KIR3DL1/S1^−^ patients (129). The influenza peptides (**Table 1**) presented in HLA-A*24:02 binds to KIR3DL1 with different affinity depending on the KIR3DL1 allele (106).

### HIV-1

NK cells play a dual role in Human immunodeficiency virus type 1 (HIV-1) infection, exhibiting both protective and negative regulatory effects (130). One month after sensitization of immunodeficient mice with viral-like particles (VLPs), containing HIV-1 antigens, injection of hepatic NK cells resulted in a recall response one more month later upon repeated stimulation with the HIV-VLPs (126). A study using humanized mouse model demonstrated that hepatic NK cells primed with HIV-Env-loaded dendritic cells (DCs) exhibited greater cytotoxicity against HIV-Env targets compared to naïve NK cells or those interacting with irrelevant DCs (2). In humans, HIV infection, particularly in individuals coinfected with hCMV, was associated with augmentation of mature CD57^+^KIR^+^NKG2C^+^NKG2A^−^ NK cells with reduced PLZF expression and increased IFNγ production and CD107a expression compared with PLZF^+^HIV^+^ NK cells and HIV^−^ controls (131). HIV-infected humans demonstrated enhanced, compared to healthy volunteers, IFNγ production and degranulation in response to the HIV Gag peptides (107). Recently, an NK cell subset co-expressing NKG2C and NKG2A was revealed to be associated with effective control of HIV replication (132).

The HIV-peptide specificity was identified for NKG2C. Clonally expanded NK cells demonstrated the specific lysis of HLA-E expressing K562 cells, loaded with the VLKYWWNLL and ILPCRIKQI HIV-Env-derived peptides and the AISPRTLNA peptide derived from HIV-Gag (107).

Several studies support the KIR3DS1 impact in HIV clearance. KIR3DS1^+^ NK cells degranulate more in response to HIV-infected CD4^+^ T cells expressing HLA-Bw4 than HLA-Bw6 (133). and inhibit viral replication in HLA-Bw4-T cells (134). Another research group confirmed the direct interaction of KIR3DS1 with HLA-Bw4 on the example of HLA-B*51 (135). A recent study revealed the ability of KIR3DS1 to bind HLA-B*57:01 presenting HIV peptides AAVKAACWW (derived from the Gag-Pol protein) and KAAFDLSFF (derived from the Nef protein) and promote the activation of KIR3DS1^+^ NK cells (101). Besides, the binding of KIR3DS1 to HLA-F was shown to play a role in KIR3DS1^+^NK cells activation in response to HIV-infected T cells (62, 136).

Peptide dependence was also observed in the interaction of inhibitory KIRs with certain HLA-I alleles. KIR3DL1 interaction with HLA-B*27:05 displaying different p24 Gag HIV peptides depends not only on peptide length but also on the amino acid situated at the penultimate position of this peptide. KIR3DL1 binds especially strongly to HLA-B*57:01/03, which presents the ISPRTLNAW peptide (105). Noninfected HLA-C*03 :04 CD4^+^ cells mostly present VIYPARISL peptide, which strongly binds to KIR2DL3; in contrast, HIV-infected CD4^+^ cells mostly present the self-peptide YAIQATETL, which interfere with KIR2DL3 binding the HLA-C allele (35).

### HCV and HBV

NK cells play a prominent role in the response to both hepatitis C virus (HCV) and hepatitis B virus (HBV). Involvement of NK cells expressing killer cell lectin-like receptor G1 (KLRG1) in both HCV and HBV infections was noted in a number of studies. KLRG1^+^ NK cells exhibited a mature phenotype, high cytotoxic activity, and increased IFNγ production compared to KLRG1^–^ NK cells (137–139).

Investigation of KIRs in HCV infection revealed that KIR2DS2 recognizes peptide LNPSVAATL (HCV helicase) in the context of HLA-C*01:02, and this recognition leads to NK cell degranulation (34). A similar peptide, IVDLMCHATF, derived from the helicase of other Flaviviridae viruses (*e.g.* Zika and dengue), can also activate KIR2DS2^+^ NK cells when presented in the context of HLA-C*01:02 (34). KIR2DS2 transgenic mice injected with HLA-C*01:02 loaded with this IVDLMCHATF peptide have also shown high NK cell degranulation levels, with cross-reactive degranulation in the presence of other HCV peptides as well (140). Another study confirmed the interaction between KIR2DS2 with HLA-C*01:02 and HLA-A*11:01 (67). Additionally, KIR2DL3 strongly binds to HLA-C*03:04 presenting the core protein-derived YIPLVGAPL peptide of HCV, and this interaction results in suppression of NK function and viral escape (104).

### SARS-CoV-2

Our and other studies have described the role of NK cells in COVID-19, showing that NK cells of patients infected with SARS-CoV-2 exhibit an activated, proliferative phenotype with clear molecular hallmarks of cytotoxic activity (141–145). Increased IFNγ production and degranulation were observed in NK cells restimulated with SARS-CoV-2 peptides post-vaccination (146) although IFNγ responses varied between donors (147), highlighting inter-individual differences in NK cell responses. Single-cell RNA sequencing studies have revealed an accumulation of NK cells sharing some traits with hCMV-associated adaptive NK cells in COVID-19-recovered donors, mostly attributed to severe COVID-19 course (148–150). These adaptive immune cells were characterized by high expression of *KLRC2* (NKG2C), low expression of *ZBTB16* (PLZF), enhanced IFNγ production and degranulation intensity, and elevated functional response under activation of type I IFN signaling (148). On the other hand, a decrease in NKp30, but not an increase in NKG2C levels was associated with lethal outcome (143). Deletion of the KLRC2 gene was shown as a risk factor for severe COVID-19 (151). Other studies as well supported the involvement of NKG2A in the interaction with SARS-CoV-2. The degranulation activity of NKG2A^+^ NK cells were inhibited in the presence of VFLVLLPLV, loaded in HLA-E (109), whereas another SARS-CoV-2 peptide VMPLSAPTL prevented binding to NKG2A, resulting in the missing-self activation of NK cells and the inhibition of viral replication (108).

Some evidence supports the role of KIRs in COVID-19. In severe patients, the proportion of cells expressing KIR2DS4 is increased and the proportion of cells that do not express any KIR receptor is reduced (152). In a recent study, we observed a significant correlation between the expression of KIR2DS4 and proliferation and IFNγ production of NK cells in response to SARS-CoV-2 peptide in SARS-CoV-2 experienced donors (153). Previously identified protective effects of the HLA-Bw4^+^ KIR3DL1^+^ combination (154) may be related to poor binding of SARS-CoV-2 peptides to HLA-Bw4, resulting in NK cell-mediated lysis of SARS-CoV-2-infected cells (155). However, the antigen-specific role of KIR^+^ NK cells is still poorly described.

### Other viral infections

An adaptive-like NK cell immune response has been observed in the context of several other viral infections. One of such observations was in a mouse model of herpes simplex virus type-2 (HSV-2) (156). NK cells were obtained from mice infected with HSV-2 one month prior and then examined in terms of their IFNγ production. Restimulation of those cells with HSV-2 antigens triggered an upregulation of IFNγ production compared to naïve NK cells (156).

Varicella-zoster virus (VZV) is another ubiquitous herpesvirus (157), and at least one study indicates that VZV infection induces long-lived memory NK cells. The injection of VZV glycoproteins into human skin of individuals who experienced chickenpox in childhood resulted in the recruitment of a large number of NK cells in blister; in contrast, no recruitment was observed after a control injection with sterile saline solution (2). Analysis of the degranulation activity of NK cells isolated from blisters has revealed enhanced CD107a production and CXCR6, NKG2D, and CD69 expression compared to donor-matched NK cells from the peripheral blood mononuclear cells (PBMCs) (2).

Epstein-Barr virus (EBV) is a gamma herpesvirus that primarily infects B cells and may turn into infectious mononucleosis (IM) at younger ages or may be latent and can eventually lead to the development of a range of malignancies, including Hodgkin’s lymphoma (158, 159). In all forms of EBV-associated disease, NK cells influence B cell infection. In one of the works, it was shown that CD56^dim^NKG2A^+^KIR^−^ NK cells accumulate in IM patients and can persist in the blood at increased frequencies for months. These low differentiated NK cells specifically recognize EBV-infected B cells and degranulate with greater intensity than CD56^bright^ or CD56^dim^NKG2A^−^KIR^+^ (160). Another study found that a similar population of NKG2A^+^NKG2C^−^KIR^−^ NK cells mediate the elimination of autologous EBV-infected B lymphoblastoid cells and produce IFNγ and CD107a (161). We demonstrated that, in EBV-seropositive individuals, latent infection was associated with an accumulation of terminally differentiated NK cells with elevated inhibitory KIRs, without enrichment of NKG2C or HLA-DR. EBV-IgG titers correlated with CD57 and KIR2DS4 levels; among KIR2DS4^+^ donors, carriage of at least one HLA-C2 allele was linked to higher EBV-IgG (162). The EBV peptide GGDPHLPTL, which is derived from latent membrane protein 1 (LMP1), strongly binds to HLA-E, and this complex interfere NKG2A/CD94 heterodimerization and activates degranulation of NKG2A^+^ NK cells. To eliminate the involvement of NKG2C in the activation process, the authors specifically sorted and tested NKG2A^+^NKG2C^−^ NK cells (33). The EBV peptide RLRAEAQVK from the EBNA3A protein can be recognized by KIR3DL2 when presented by HLA-A*03:01 or HLA-A*11:01 (64). However, the signals produced by KIR3DL2 in this context have not been described. The activating receptor KIR2DS4 can bind to HLA-A*11 and may possibly recognize that same EBNA3A-derived peptide (69).

The involvement of HLA-related receptors in recognition of viral peptides was investigated in the following infections. Simian immunodeficiency virus (SIV) causing the acute infection of rhesus macaques, was shown to be hampered by NK cells (163, 164). Splenic and hepatic NK cells from SIV-infected macaques demonstrated enhanced lysis of Gag- and Env-pulsed DCs through NKG2A and NKG2C action (165). Besides, NK cells were shown to control the SIV infection of African green monkey (AGM). SIVagm infection induced the expansion of terminally differentiated NK cells with an adaptive transcriptional profile, while without upregulation of NKG2C expression and increased MHC-E-restricted cytotoxicity in response to SIV Env peptides. Autologous NK cells strongly suppressed viral replication in the presence of the SIVagm-ENV IGIVVIVKL peptide presented on SIV-infected CD4^+^ T cells, whereas substitution with the SIVmac peptide NQLLIAILL allowed the virus to escape NK cell-mediated suppression (166).

Vaccinia virus (VACV) belongs to the Poxviruses, which cause a mild infection in humans and is used as a base for vaccine creation (167, 168). KIR2DS2 strongly binds to HLA-A*11:01 with loaded MLIYSMWGK peptide, derived from VACV membrane protein A14 (MpA14) (100). Human adenoviruses (HAdVs) are nonenveloped double-stranded DNA *Mastadenoviruses*, which infect many cell types and can cause diverse diseases including respiratory tract infections, gastroenteritis, and hepatitis (169, 170). HAdV infection leads to decreased expression of HLA-A and -B molecules, while amounts of HLA-F start to rise. KIR3DS1^+^ NK cells achieve faster clearance of HAdVs (171), and KIR3DS1 generally confers protection against several other viral infections.

Collectively, approaches for detecting antigen-specific NK cells vary across studies, but, overall, these efforts contribute to a better understanding of the ability of NK cells to form relatively antigen-specific memory.

Thus, the role of NKG2 and KIR receptors has been shown for various viral infections that can persist in the body for a long time, cause chronic disease or regularly circulate in the human population, often with co-infection with hCMV, which leads to accumulation of NKG2C+ NK cells. The interaction of KIRs with viral peptides presented in HLA-I can lead to specific activation of NK cells. NKG2C may enhance NK cell activation through the interaction with HLA-E in complex with a peptide originated from the leader sequence of HLA-G, the increased expression of which often accompanies viral infections (172–174). Hence, in these viral infections, the NKG2C-HLA-E interaction system, even in the absence of an HLA-E-binding specific viral peptide, can support the activation and proliferation of NK cells, which were already primed by the specific interaction of KIR with viral peptides presented in HLA-I.

### Bacteria

Although NK cells primarily protect against viral diseases and cancer (32, 175, 176), they also have an important role in fighting bacterial infections (177). Several studies highlight the adaptive-like response of NK cells to bacteria. For instance, in *Ehrlichia muris*-infected mice, memory-like NK cells were induced, and transferring these NK cells to RAG-deficient mice protected them against infection and death upon re-exposure to the same strain, unlike naive NK cell transfers, which resulted in lower survival (178).

*Mycobacterium tuberculosis* (Mtb) is adept at evading immune responses, but NK cells are vital in controlling Mtb infection (179). Pleural fluid-derived memory NK cells from Mtb-infected patients typically exhibit expression of a memory-associated marker CD45RO (180) and produce more IL-22 in response to Bacille Calmette-Guerin (BCG) vaccine stimulation compared to CD45RO^−^ NK cells or those from PBMCs (181). CXCR6^+^ NK cells from Mtb-infected patients show higher IFNγ production in response to HN878 Mtb antigens than CXCR6^−^ NK cells or those from healthy individuals (182). BCG-vaccinated mice exhibited an increase in KLRG1^+^ memory-like NK cells, the transfer of which to naïve mice resulted in enhanced IFNγ production in the lungs following *Mtb* infection compared to buffer-vaccinated controls (183). Similarly, NK cells maintained elevated IFNγ responses five weeks after BCG re-vaccination, indicating recall potential (184).

KIRs also play a significant role in NK cell-mediated defense against bacteria. Sim et al. searched for bacterial peptides with sequences similar to the above-mentioned IIDKSGAWV peptide (68), which facilitates KIR2DS4 binding when presented by HLA-C*05:01. They tested 14 recombinase A (RecA)-derived peptides for their ability to facilitate formation of a stable complex between these two proteins and observed the strongest binding with the IVDKSGAWF peptide derived from *Campylobacter jejuni*. This interaction resulted in robust degranulation of CD56^dim^KIR2DL1/S1^-^ NK cells. They also identified ~100 other bacterial peptides that can potentially activate KIR2DS4^+^ NK cells (68).

These data show how bacterial infections can induce the formation of memory-like NK cells, and the involvement of KIRs in the recognition of bacterial peptides supports the antigen-specific role of NK cells in eliminating such infections.

## Antigenic specificity of NK cell clones

Different research groups have proven the specific recognition of viral and bacterial peptides loaded into HLA molecules by KIRs and NKG2 receptors (**Table 2**). Besides, memory responses of NK cells have been observed under different infection conditions (122, 139, 153, 178, 183). The formation of cellular memory with diverse specificities implies stable clonal expansion to facilitate antigen-specific or antigen-adaptive responses. In this context, clonal expansion refers to the enhancement in the number of effector cell subpopulations that are best suited to combat a specific pathogen. The molecular and cellular mechanisms underpinning clonal NK cell specificity still remain unclear.

**Figure 2:**
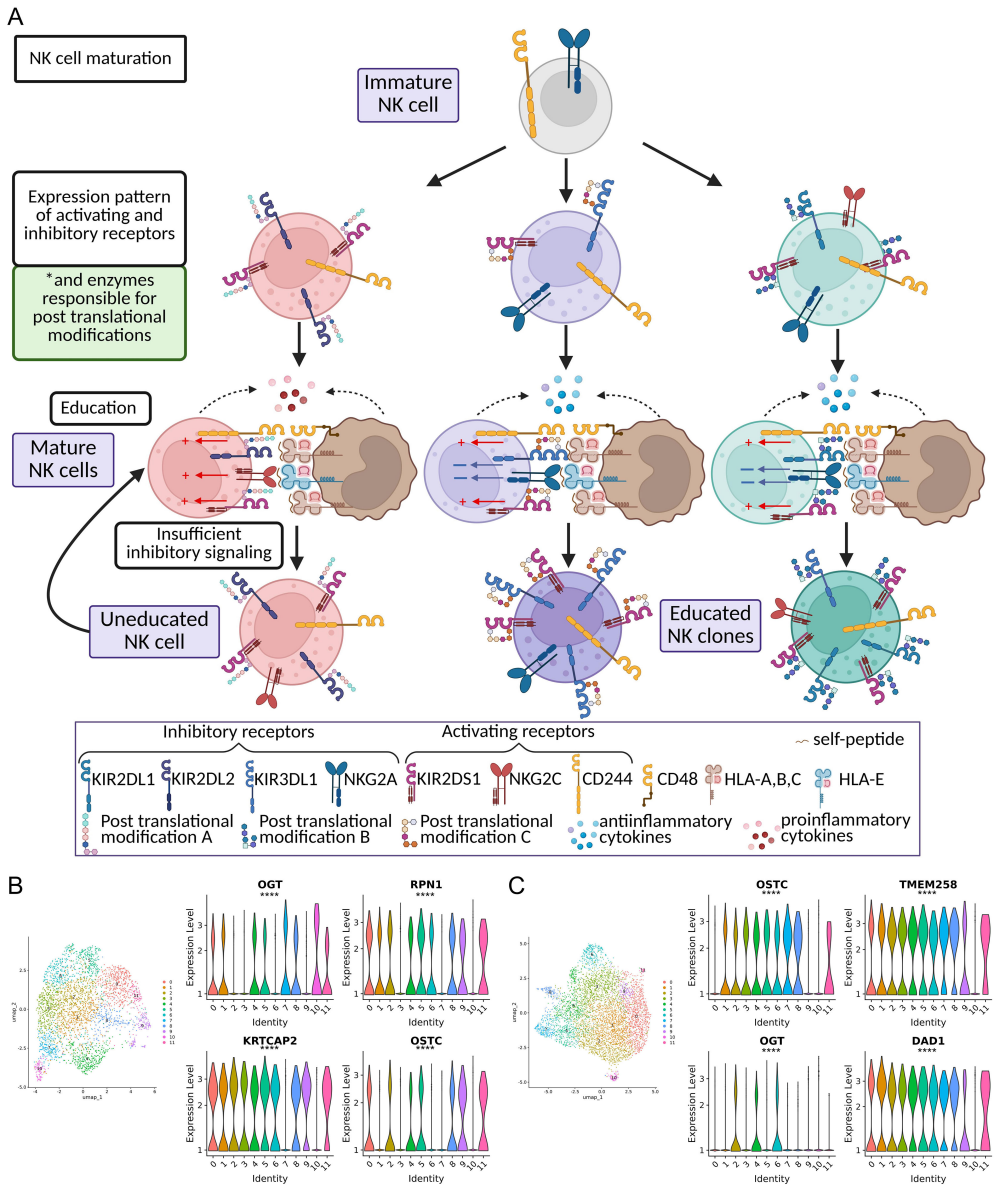
Post-translational modification hypothesis, upon NK cell maturation and education processes. **(A)** At top is an immature peripheral blood NK cell expressing inhibitory NKG2A receptors and the CD244 receptor from the SLAM family. The cell undergoes random selection of the activating and inhibitory receptor expression pattern (mainly from the KIR family), and we hypothesize that this maturation process is also coupled with a random selection of glycosyltransferases expression, that determines the pattern of post-translational modification to those receptors. The second row presents three cells expressing different inhibitory KIRs and activating receptor, KIR2DS1, with different glycosylation patterns. The next row shows the education process, during which NK cell receptors interact with self-pMHC complexes. Recognition of self-pMHC by inhibitory KIR. These interactions, together with NKG2A-HLA-E, CD244-CD48, and weak KIR2DS4-HLA-I interactions calibrate activation thresholds, yielding either educated/functionally competent (bottom right) or uneducated states (bottom left). Simultaneous cytokine exposure further stimulates successful NK cell education and maturation (bottom right). **(B, C)** UMAP plot of scRNAseq data available at GSE184329 and Violin plots reflecting the expression level of some differentially expressed glycosyltransferases (ENSG00000147162 (OGT), ENSG00000163902 (RPN1), ENSG00000163463 (KRTCAP2), ENSG00000198856 (OSTC), ENSG00000134825 (TMEM258), ENSG00000129562 (DAD1)) among NK cell clusters from two different samples.

Clues to understanding NK antigenic specificity may be found in the processes of the NK cell clones maturation and education, in an indirect analogy with the positive and negative selection of T cells in the thymus (3, 10, 185). NK cells lack the V(D)J recombination process that produces such enormous diversity in terms of distinct T cell receptor protein structures. Instead, the roots of NK cell repertoire breadth lie in the combinatorial diversity of expressed receptors. During NK cell maturation, the expression pattern of activating and inhibitory NK cell receptors is defined for each NK cell. Initially essentially random, this pattern can be further imprinted in a clonal progeny of this particular cell (10, 111). The subsequent education process arises through the interaction of NK cell receptors with self-peptide-laden HLA molecules (186, 187). Thereby, education leads to the ability of NK cells that have received the corresponding inhibitory signal to respond robustly while leaving others in an uneducated state (**Figure 2**). This tuning is context-dependent and can be modulated by inflammatory cytokines and tissue cues (11, 22).

The extensive allelic variation of KIRs, coupled with their random expression, creates a vast repertoire of unique clonal populations of NK cells (**Figure 2**) (188). The theoretical diversity of the NK cell repertoire is estimated to encompass > 1,000,000 variants of NK cells in the individual (189). However, this diversity does not fully explain the differential affinity of KIR-pMHC interactions and the underlying prerequisites for peptide-specific action of NK cell clones.

For several NK cell receptors, it has been established that posttranslational modifications can modulate the affinity of receptor-ligand interactions. In particular, glycosylation plays a key role in modulating NK cell activity during infection, facilitating a flexible immune response. For instance, the N-linked glycosylation of the 2B4 co-receptor on NK cells is vital for its affinity for the ligand CD48 (190). Conversely, sialylation of 2B4 diminishes this affinity. In another example, glycosylation sites on the CD16 antibody-binding Fcγ receptor have been delineated (191), revealing that N-glycosylation of CD16 on NK cells modulates its interaction dynamics with IgG (192).

Further, mutagenesis studies on the ectodomain of NKp30 have identified three distinct N-linked glycosylation sites, where varying glycosylation patterns (*i.e.*, mono-, di-, or triglycosylation) yield differential binding affinities and downstream signaling behaviors (193). Enhancement of the function of key NK cell receptors through changes in their glycosylation may promote clonal NK cell activation, initiated by the peptide-dependent interaction of activating KIRs with their ligands, acting as a kind of second signal. Moreover, KIRs are also known to be subject to post-translational modifications including glycosylation and phosphorylation (194); however, a significant knowledge gap remains regarding the details of KIR post-translational modification.

In our study of SARS-CoV-2-experienced donors, we observed the upregulation of the glycosyltransferase *DPAGT1*, accompanied by co-upregulation of KIR2DS4 in NK cell samples stimulated with SARS-CoV-2 peptides compared to control samples (153). In order to assess the diversity of gene expressions involved in glycosylation we analyzed publicly available scRNA-Seq datasets (149). The expression patterns of glycosyltransferases distinctly varied across NK cell clusters, indicating their different cellular glycosylation landscape, which indirectly supports our hypothesis (**Figures 2B, C**). While the upregulation of a glycosyltransferase cannot directly confirm the modification of KIRs or other receptors, the observed variability among clusters highlights a potential avenue for further exploration of this concept.

## Impact of KIR glycosylation hypothesis

**Figure 3:**
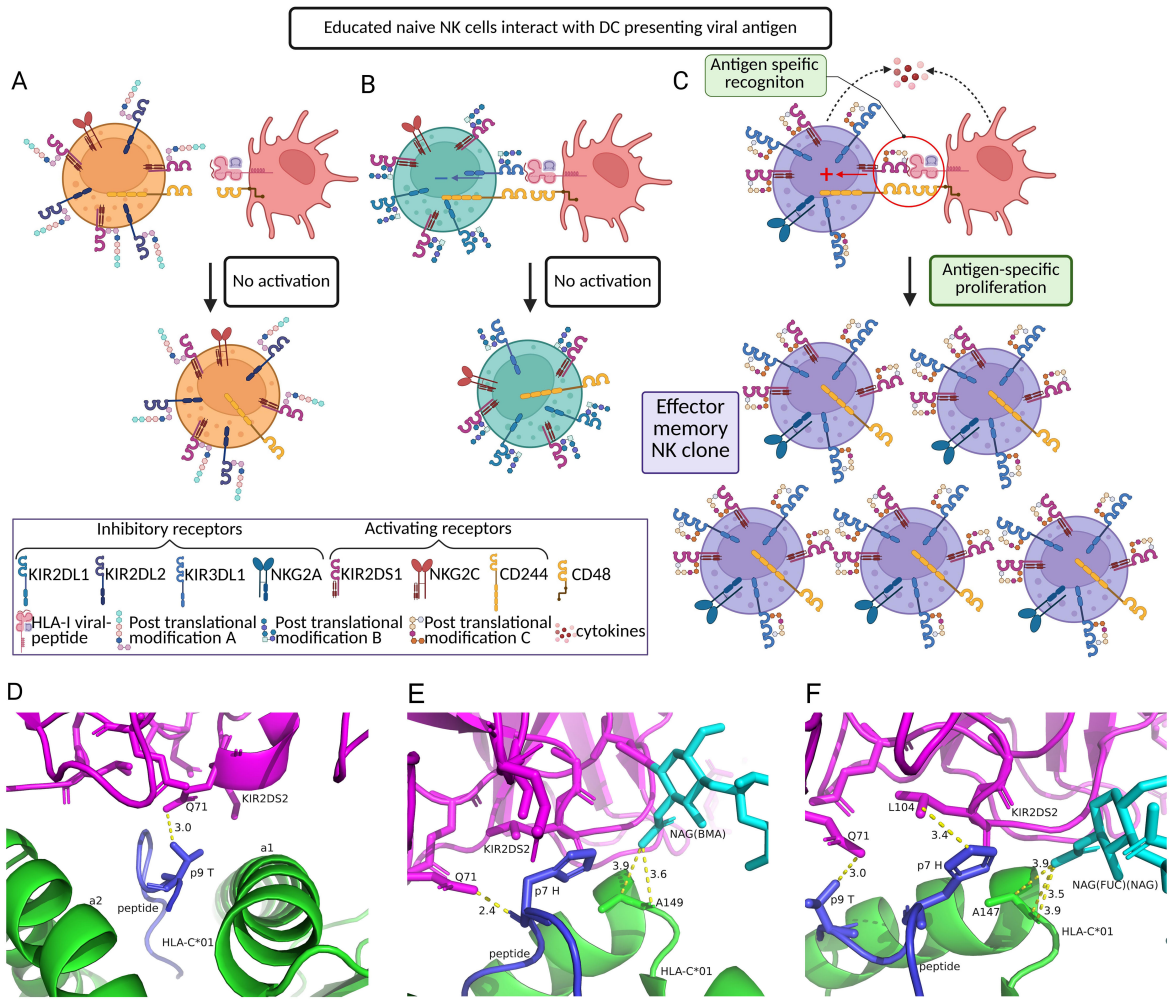
NK cell clone priming in response to viral infection. Several aspects of the NK cell surface receptor profile influence the outcome after encountering a viral peptide presented by HLA-I on a dendritic cell. **(A, B)** NK cells expressing inappropriately glycosylated activating receptor will fail to engage with the target **(A)**, as will cells with receptor combinations that achieve suboptimal peptide-HLA recognition, resulting in dominant inhibitory signals from the KIR2DL1 receptor and no activation event **(B)**. **(C)** Cells with the appropriate activating receptor and receptor glycosylation profile will engage with the target and undergo successful activation, forming a memory NK cell pool that can respond rapidly upon re-encountering the same pathogen. **(D-F)**. The AlphaFold3 prediction of the interaction between KIR2DS2-IVDLMCHATFpeptide-HLA-C*01 complex **(D)** without modification **(E)** 190N-linked *N*-acetyl-β-D-glucosamine (NAG) with β-D-mannose (BMA) modification **(F)** 190N-linked NAG with α-L-fucose (FUC) and NAG modification, which are colored in cyan. The polar contacts formed within the interaction interface are highlighted by a yellow dotted line. The distances between aminoacids are in Å.

Glycosylation sites for various KIRs were predicted over 20 years ago, and these sites have been shown to be located relatively far from the binding groove involved in HLA-KIR interactions (194, 195). However, recent research on macaques provided further insights into the functional relevance of glycosylation (196). The glycosylation of domain 0 (D0) of KIR3DL1 in two N-linked sites contributes to the specific interaction with exact HLA alleles (196). Alternatively, activating KIR functional changes may result from the specific glycosylation. An example of bioinformatic prediction (197, 198) of the KIR-HLA complexes depending on the expressed glycosyl by in silico modeling of the KIR2DS2-IVDLMCHATFpeptide-HLA-C*01 interaction interface revealed the difference in polar contacts between KIR2DS2 and peptide regardless of the addition of the glycosyl modifications into the N-linked glycosylation site of the KIR2DS2 (**Figure 3**). These glycosyl modifications formed additional contacts with HLA-C*01, potentially enhancing the overall avidity of the interaction. Furthermore, different glycosylation patterns differentially altered the interaction between KIR2DS2 and the peptide. Based on those insights, such modifications may change receptor conformation and thereby affect their affinity for distinct pMHC complexes.

We hypothesize that these intricate molecular changes could play a role in mediating the semi-antigen-specific responses of NK cells. We propose that the random selection of the expression pattern for enzymes involved in post-translational modification occurs together with the random selection of KIRs and other NK receptor expressions (10, 111), and exploits similar epigenetic programming machinery.

**Figure 4:**
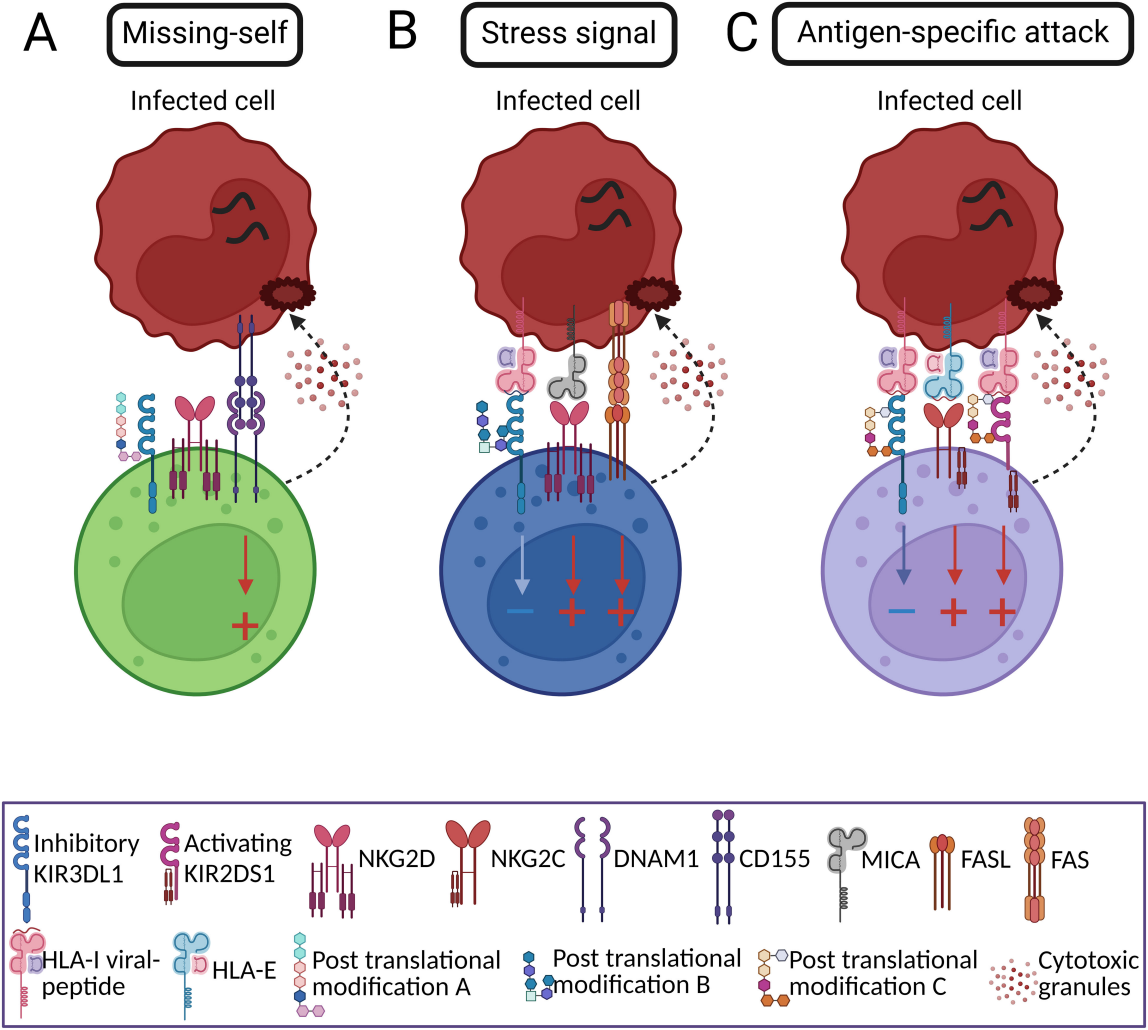
Examples of NK cytotoxic activity against virus-infected cells. **(A)** Missing-self recognition of target cells that do not express the HLA-I molecules induce NK cell activation. **(B)** Stress signal recognition of activating ligands that are expressed on target cells by NK cell receptor NKG2D (ligand MIC-A), and FAS ligand (FAS receptor) overcome HLA-I-mediated inhibitory signaling resulting in NK cell activation. **(C)** Antigen-specific recognition by the effector memory NK clone through interaction between glycosylated activating KIR and HLA-I presenting viral peptide.

There are more than 200 glycosyltransferase genes, of which 173 are involved in protein glycosylation (199). Distinct combinations of these enzymes may establish preferred patterns of receptor posttranslational modification, thereby affecting their average affinity to particular pMHC complexes and creating fairly unlimited diversity of NK cell receptor surfaces that may preferentially interact with distinct cognate antigens or distinct MHC subtypes (**Figure 2**). Glycosylated patterns can thus represent clonally characteristic features along with the inhibitory and activating receptors patterns. Upon interaction with a putative target cell, NK cells expressing activating KIRs with glycosylation patterns that confer higher affinity to a particular pMHC would undergo more robust proliferation and thereby ‘win’ in the ongoing clonal competition, analogous to the priming of naive T cells expressing a TCR specific to a particular antigen followed by clonal competition (176, 200) (**Figure 3**). During subsequent pathogen exposure events, these effector NK clones would be able to perform semi-antigen-specific killing of infected cells (**Figure 4**). This model suggests a plausible explanation for the antigen-specific character of NK cell memory previously observed in multitude studies (33, 137, 156, 180).

Notably, infections can induce significant alterations in the host’s cellular glycosylation machinery, often driven by inflammatory cytokines (201). For instance, pro-inflammatory cytokines like TNF-α have been shown to directly influence the expression of glycosyltransferases, leading to observable shifts in the glycosylation patterns of plasma proteins and immune cell surfaces (202). The treatment of HIV-infected patients with IFN-a induced an increase in fucosylation of NK cells, which positively correlated with the NK cell functionality (203). Helicobacter pylori infection of gastric epithelial cells resulted in differential regulations of some glycosylation-related genes (such as MUC20 and REG4), underscoring infection-driven remodeling of glycan pathways (204). Changes in glycosylation associated with immunoregulatory cytokine networks and inflammation may contribute to NK cell adaptation to viral load and infection control.

## Conclusion

This review explores recent insights into NK cells’ ability to mount adaptive, clonal, and antigen-specific responses to intracellular infections. NK cells can form diverse long-lived memory clones, which can rapidly counter secondary infections. Similarly to the T cell world, the phenotype and action programs of such NK cell clones may vary depending on the nature of foreign agents (205, 206). The combinatorial effect of clone-specific expression of HLA-related receptors, potentially multiplied by the combinatorial effects of differential expression of enzymes involved in their post-translational modification, enables near-unlimited clonal diversity. This allows NK cells to perform clonal antigen-specific immune responses, albeit with a less deterministic pattern of recognizing receptor surface than genetically recombined TCRs.

Despite significant advances in understanding adaptive and memory functions of NK cells, many aspects of their antigen-specific responses remain unclear. The apparent lack of similarities between NK cell clones responding to specific infections in different individuals currently hampers investigation of the basics for their antigenic specificity or semi-specificity. Further investigation with cutting-edge techniques, including single-cell transcriptomics and ATAC-Seq, with a possible focus on glycosyltransferases and proteomic analysis of NK cell clones, along with epigenetic studies, could shed light on the mechanisms underlying antigen-specific NK cell memory. These insights could redefine NK cells’ adaptive roles and advance NK-based immunotherapies for infections and cancer.
